# A Case of Cefepime-Induced Neurotoxicity: Renal Function Missing in Action

**DOI:** 10.7759/cureus.13368

**Published:** 2021-02-16

**Authors:** Brooke P Lichak, Omolara Lawal, Hyma V Polimera, Ashwani Garg, Gurwant Kaur

**Affiliations:** 1 Internal Medicine, Penn State Health Milton S. Hershey Medical Center, Hershey, USA; 2 Neurology, Penn State Health Milton S. Hershey Medical Center, Hershey, USA; 3 Nephrology, Penn State Health Milton S. Hershey Medical Center, Hershey, USA

**Keywords:** cefepime-induced neurotoxicity, cefepime, neurotoxicity, encephalopathy, acute kidney injury, toxicity, seizure, acute interstitial nephritis, acute tubular necrosis

## Abstract

Cefepime is a renally excreted, fourth-generation cephalosporin used in the treatment of severe abdominal, skin, soft tissue, and urinary tract infections due to its broad-spectrum coverage. Cefepime-induced neurotoxicity is a rare but serious side effect that has increased in recent years likely due to increased antibiotic use, increased drug resistance, and increased symptom recognition. While decreased glomerular filtration rate is an important risk factor for developing elevated serum cefepime levels, recent literature has suggested that a significant proportion of patients with normal renal function can also develop neurotoxicity from cefepime. Here, we present a case of cefepime-induced neurotoxicity to demonstrate the importance of monitoring mental status changes in all patients being treated with cefepime.

## Introduction

Cefepime-induced neurotoxicity has a wide range of symptoms including impaired consciousness, encephalopathy, myoclonus, seizures, and coma [1]. Risk factors include decreased glomerular filtration rate (GFR), high drug dosing, and dysfunction of the blood-brain barrier. The primary proposed mechanism of cefepime-induced neurotoxicity is competitive inhibition of γ-amino butyric acid receptors in the central nervous system [2]. This suppression of the primary inhibitory receptor in the central nervous system likely lowers the threshold of excitation which subsequently can result in seizures or global encephalopathy. Due to the increasing incidence of cefepime-induced neurotoxicity and prevalence in healthy individuals, it is imperative for physicians to have a high index of suspicion for neurotoxic effects when treating patients with cefepime. By closely monitoring a patient’s renal function, adverse consequences can be recognized quickly and managed appropriately. This article was previously presented as a poster at the Society of Hospital Medicine South Central Pennsylvania Chapter annual meeting on October 17, 2020.

## Case presentation

A 59-year-old female with a history of neuromyelitis optica with baseline paraplegia and neurogenic bladder on chronic foley presented to the emergency department with altered mental status.

**Figure 1:**
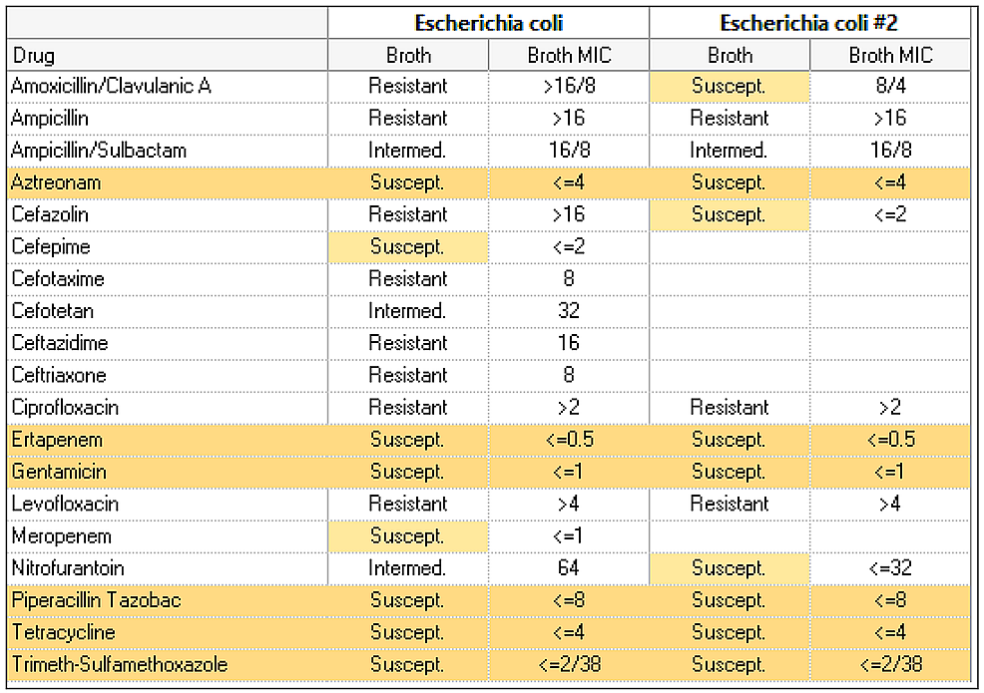
Urine culture and sensitivity report on first admission.

She was recently admitted to the hospital with septic shock requiring pressors for one day secondary to resistant *Escherichia coli *urinary tract infection and acute kidney injury (AKI) with elevated creatinine of 1.4 mg/dL (baseline of 0.6-0.7 mg/dL approximately five months ago) and decreased creatinine clearance (CrCl) of 36 mL/min. Her renal function quickly improved on day two of admission to a creatinine of 0.7 mg/dL and CrCl of 56.5 mL/min. She was initially started on intravenous (IV) ceftriaxone and later changed to IV cefepime due to her urine culture, sensitivity pattern, and renal recovery (Figure 1). A PICC line was placed and the patient was discharged with IV cefepime. She had completed a 10-day course of cefepime just one day prior to the current admission.

**Figure 2:**
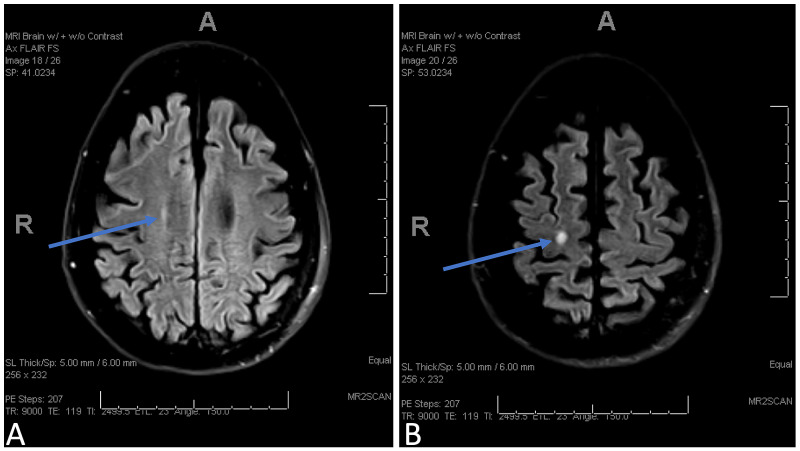
Axial FLAIR MRI. (A, B) Right centrum semi-ovale with hyperintense demyelinating lesion without enhancement (blue arrows). FLAIR, fluid-attenuated inversion recovery; MRI, magnetic resonance imaging

During this admission, initial vitals were temperature 35.8ºC, heart rate 68 beats per minute, respiratory rate 19 breaths per minute, and blood pressure 81/54 mmHg. Her systolic blood pressures were in 80s for the last two to three months prior to admission and was maintained on home midodrine. This was attributed to autonomic dysfunction secondary to neuromyelitis optica after extensive workup. Examination was notable for expressive aphasia and altered mental status. She was also found to have AKI with elevated creatinine of 2.19 mg/dL (baseline 0.6 mg/dL), blood urea nitrogen of 68 mg/dL (baseline 7 mg/dL), and estimated glomerular filtration rate (eGFR) of 23 mL/min/1.73 m^2^ (baseline >60 mL/min/1.73 m^2^). Electrolyte levels were serum sodium 145 mmol/L (baseline 141 mmol/L), serum potassium 4.4 mmol/L (baseline 4.3 mmol/L), serum chloride 108 mmol/L (baseline 104 mmol/L), and serum bicarbonate 18 mmol/L (baseline 26 mmol/L). Her urine output was decreased to 600 mL in 24 hours. Brain computed tomography did not show any acute intracranial findings. Magnetic resonance imaging of the brain showed an interval increase in the size of the right centrum semi-ovale and a hyperintense demyelinating lesion without enhancement which ruled out an acute demyelinating process (Figure 2).

**Figure 3:**
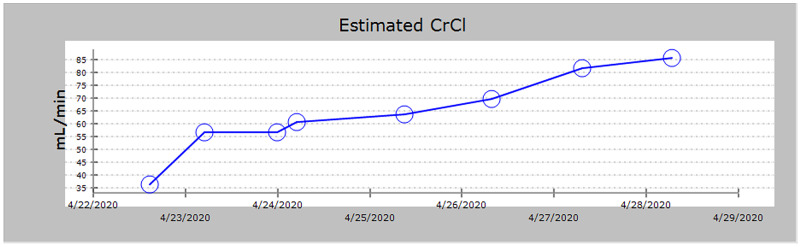
Trend of CrCl (mL/min) during first admission. CrCl, creatinine clearance

She continued to become more lethargic over the next few hours and developed jerking of the left upper extremity and face. Medical management to treat the seizures was partially successful. Serum cefepime levels were measured and found to be elevated at 295 mg/L. After three hours of hemodialysis, cefepime levels improved from 295 mg/L to 70 mg/L. She was then started on continuous renal replacement therapy (CRRT) for ongoing clearance of cefepime. Serum cefepime level the next day was 17 mg/L. Her mental status continued to improve and CRRT was stopped 48 hours after initiation. Figure 3 shows the trend of the patient’s renal function during her first admission.

**Figure 4:**
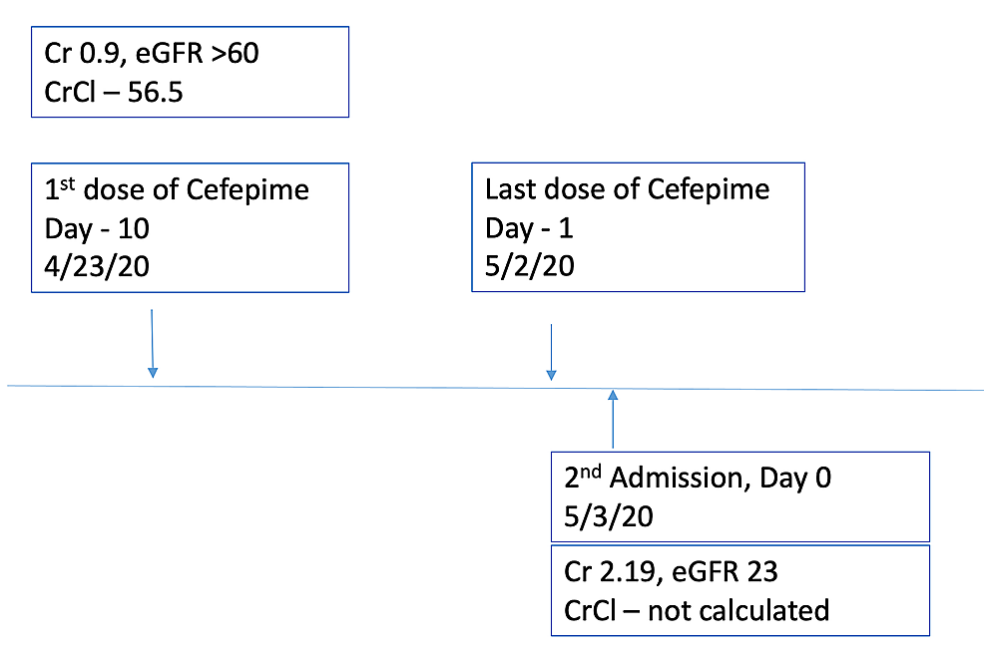
Timeline of creatinine and CrCl in relation to admission. CrCl, creatinine clearance (mL/min); Cr, Creatinine (mg/dL); eGFR, estimated glomerular filtration rate (mL/min/1.73 m^2^)

Figure 4 shows the timeline of renal function in relation to her current admission. She did not require additional dialysis while hospitalized.

## Discussion

Elevated serum cefepime level is an essential predictor of neurotoxicity, with a trough >20 mg/L associated with a five-fold higher risk for neurologic events [3]. For this reason, a trough >20 mg/L should generally be avoided. Predisposing factors of cefepime-induced neurotoxicity include excessive dosing, reduced renal function, increased cerebral penetration, and increased serum antibiotic levels [4].

While it is stated that less than 1% of patients experience neurotoxic side effects [5], recent literature has noted up to a 10-fold increase in the incidence of cefepime-induced neurotoxicity [6]. The increased incidence is likely due to increased antibiotic use, increased drug resistance [7], and increased symptom recognition [6]. A systematic review published in 2017 showed that up to 15% of all intensive care unit patients treated with cefepime experienced neurotoxic effects including encephalopathy, seizures, and coma, as well as subtler side effects such as depressed consciousness [1]. The study also showed that 26% of the patients experienced neurotoxic symptoms despite proper dosing and 20% of patients did not frequently experience renal dysfunction prior to the neurotoxic episode [1].

This also highlights the importance of antibiotic stewardship and careful monitoring of signs of cefepime-induced neurotoxicity while following renal function. It involves dosing antibiotics carefully based on estimated CrCl. This is especially valuable in hospitalized patients with AKI where eGFR might not be a reliable indicator of true eGFR, as eGFR equations are applicable for steady state. Kinetic eGFR was not calculated in this case. Her underlying chronic kidney disease, as revealed by the biopsy, likely played a significant role in predisposing to frequent AKIs (current and previous admission) and reduced renal clearance of the drug, and hence, precipitating acute cefepime neurotoxicity.

**Figure 5:**
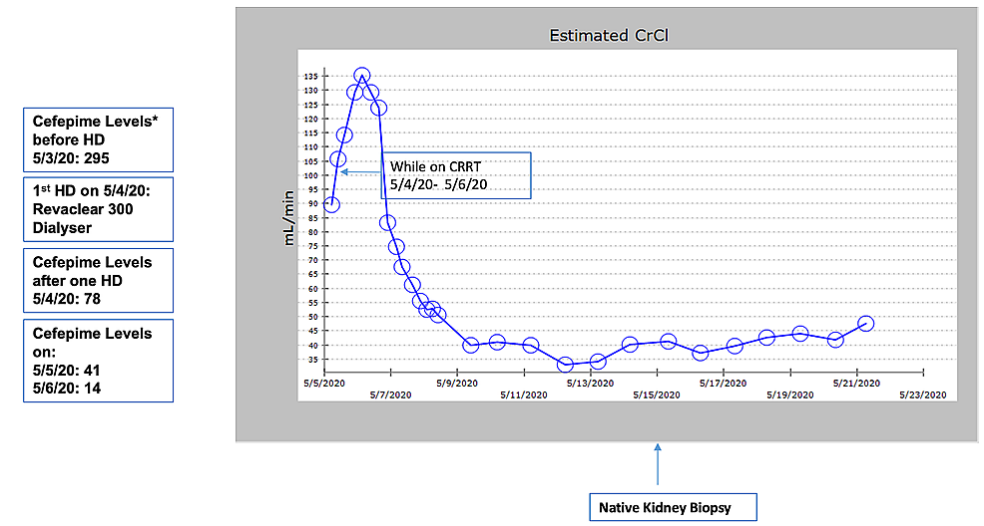
Trend of CrCl during current admission. *All cefepime levels are in mg/L HD, hemodialysis; CrCl, estimated creatinine clearance (mL/min); CRRT, continuous renal replacement therapy

AKI is likely explained by acute tubular necrosis secondary to septic shock with need of vasopressors. Improvement in her neurological status after dialysis with reduction in serum cefepime level puts our diagnosis on a firm footing. Figure 5 shows the timeline of the events of dialysis in relation to cefepime levels.

## Conclusions

To decrease the incidence of cefepime-induced neurotoxicity, treating patients with antibiotics such as ceftriaxone or cefotaxime can be considered due to their hepatic excretion. If cefepime must be administered, electroencephalogram (EEG) should strongly be considered in any patient with an acute change in mental status. This highlights the need to be vigilant in recognizing clinical signs and symptoms early. By monitoring the patient’s mental status, utilizing EEG, measuring cefepime levels, and tracking renal function, adverse consequences can be recognized quickly and managed appropriately. Renal function should be monitored closely with monitoring of urine output, CrCl, and kinetic eGFR. In patients with severe neurotoxic symptoms, hemodialysis may be employed to rapidly reduce antibiotic levels in the serum and antiepileptic drugs should be administered. Dosing of antiepileptic drugs should be adjusted in relation to the timing of dialysis to maintain therapeutic levels.
